# The first absolute gravity and height reference network in Sicily

**DOI:** 10.1038/s41597-024-03177-4

**Published:** 2024-04-08

**Authors:** Filippo Greco, Giovanna Berrino, Federica Riguzzi, Augusto Mazzoni, Matteo Amendola, Daniele Carbone, Danilo Contrafatto, Gino Dardanelli, Mauro Lo Brutto, Antonino Maltese, Alfio Messina, Luca Mirabella, Giuseppe Ricciardi, Luca Samperi

**Affiliations:** 1 INGV, sez. Osservatorio Etneo, Catania, Italy; 2 INGV, sez. Osservatorio Vesuviano, Napoli, Italy; 3 INGV, sez. Osservatorio Nazionale Terremoti, Roma, Italy; 4 DICEA, Università La Sapienza, Roma, Italy; 5 Università degli Studi di Palermo, Palermo, Italy

**Keywords:** Geophysics, Design, synthesis and processing

## Abstract

The purpose of this work is to provide the methodological and instrumental framework for the establishment of a new absolute gravity and height reference network in Sicily. The aim of the network is to contribute to the new reference systems in the Italian area, useful for the scientific and technological activities related to the gravity field and to the proper definition of a modern height system in this region. The network is composed of 5 stations, evenly distributed to form a large mesh, which roughly covers the entire Sicily. Since four of the five selected stations were measured also in the 1990s, it was also possible to evaluate whether long-term gravity changes occurred at these sites (basic requirement for a reference network) and check the long-term ground deformation patterns, using data from the closest GPS/GNSS stations. The observed gravity changes over a time interval of about 30 years at the absolute stations and in the surrounding areas, confirm the long-term stability of the selected areas/sites.

## Background & Summary

The Italian area is affected by ground deformation and mass transfer processes, both of geophysical and human origin, acting at very different temporal scales and significantly modifying the gravity field over time.

The current Italian gravity database contains gravimetric measurements taken on land and sea by various Research Institutions and Services. Available data were acquired at different epochs in the past 50 years, with different instruments and with non-uniform operational procedures of data acquisition and analysis, implying that, in most cases, data are not comparable with each-other. Due to this inhomogeneity, the database cannot be used to produce an updated image of the Italian gravity field.

On the other hand, the availability of reliable geodetic reference systems is essential for measuring and interpreting global change effects, for monitoring sea level variations and climate changes, for natural disaster management and to provide reliable information for decision makers.

This work aims at implementing the absolute gravity and height reference networks in Sicily. These networks will be part of the new Italian Reference Gravity and Height Networks (G0-H0), currently under development in the frame of the 2019–2022 INGV Project Pianeta Dinamico (Task S2) and the PRIN2020 project (funded by MUR, the Italian Ministry of University and Research). These are fundamental infrastructures for all the scientific and technological activities involving measurements of the gravity field and a proper definition of a modern height system in Italy. These activities presented in the following sections are in line with the actions promoted by the International Association of Geodesy, that during its 2015 General Assembly approved two resolutions: the establishment of the new global gravity network (to replace the obsolete International Reference Gravity Network IGSN71^1^) and the establishment of the global physical height network, the International Height Reference System/Frame (IHRS^2^/IHRF). These resolutions introduced new international standards on gravity and height that are going to be adopted by national agencies all over the world^3,4^.

The existing absolute gravity network was updated by adding and/or remeasuring gravity stations through unique and uniform operational procedures of data acquisition and analysis, already applied in different areas^5–7^.

Remarkable impacts of the results are expected in related sciences (e.g. Geodesy, Oceanography, Geophysics, Metrology) and in their technological applications. Particularly, the new results will allow a new geoid estimation, coherent with the IHRS/IHRF standards.

### Network description

**Fig. 1:**
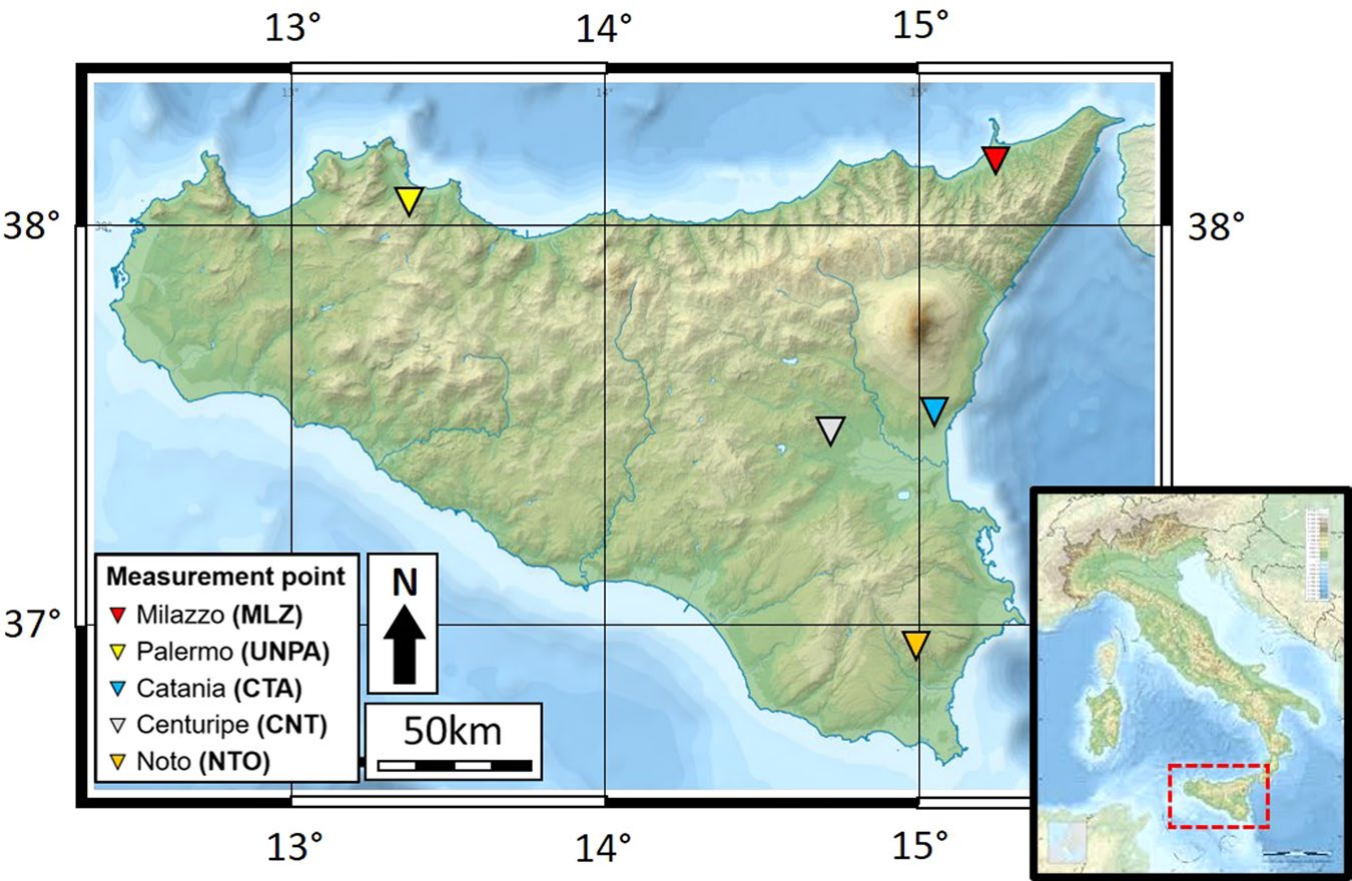
Location map of the absolute gravity and height stations constituting the reference network in Sicily. Measurement locations are Palermo and Milazzo (in the North), Centuripe (in the center), Catania and Noto (in the east).

In order to realize the gravimetric and height reference network in Sicily, we selected 5 stations (Fig. 1), including the sites of previous absolute measurements^8^, in order to control any potential changes of the gravity values or to confirm the long-term stability: Palermo and Milazzo (in the north west and north east, respectively), Centuripe (in the center), Catania and Noto (in the eastern and south-eastern part). Of these, 3 stations (Centuripe, Milazzo and Noto) were already measured in the 1990s^8^; 1 station (Palermo) is new and 1 station (Catania) is located in the gravity laboratory of the Osservatorio Etneo (INGV), where since 2007 absolute gravity measurements have been carried out to check the performances of the Micro-g LaCoste FG5#238 transportable absolute gravimeter^6,7,9–12^.

The latter instrument, described below, was used to perform all the absolute gravity measurements presented in this study. At each site, measurements of the local vertical gravity gradient were also carried out, in order to transfer the measured g value at the ground, or at any chosen reference height. Furthermore, the gravity differences (Δg) between inside absolute stations and outside satellite stations were measured. The vertical gravity gradient and the gravity differences between absolute and satellite stations were measured using the Autograv Scintrex CG-6 relative gravimeter.

A decisive constraint in the choice of the sites was the availability of indoor space with characteristics suitable for housing the absolute gravimeter. Each reference point is materialized with a stainless steel marker reporting, in capital letters, the acronym of the site name (CTA: Catania, CNT: Centuripe, MLZ: Milazzo, NTO: Noto, UNPA: Palermo).

In addition, particular attention was paid to guarantee possible future reoccupations, ensuring low levels of noise to achieve data with high precision and accuracy.

## Methods

### Gravity

The absolute gravity measurements were performed by Micro-g LaCoste FG5#238 (owned by Eni S.P.A., loan for use and managed by INGV-OE).

This instrument is mainly designed for measurements in laboratory or quasi-laboratory conditions; it is not well-suited for portable use on the field (it weighs approximately 300 kg); nevertheless, field measurements can be performed, if specific precautions are taken^6,7,9–17^.

The FG5 needs to be powered by AC and features an operating temperature range of 10 to 30 °C. It operates by using a ballistic free-fall method where a test mass (retro-reflective corner cube) is dropped vertically by a mechanical device (drug-free cart) inside a vacuum dropping chamber; the instrument is furnished of an ion pump which runs continuously in order to ensure the vacuum. The free-fall trajectory of the corner cube is monitored very accurately using a laser interferometer, allowing the precise determination of the absolute g value.

During the configuration phase, the numbers of sets and drops is defined. Measurements are generally taken during the night, when anthropic noise is weaker and higher-quality data can thus be collected.

The g measurement is subsequently processed using a dedicated software (“g”), provided by the manufacturer of the FG5, that undertakes the automatic data acquisition, the real time processing and data storage.

The resulting g values are automatically corrected by the software for the effects of the solid-earth tides, ocean tide loadings, polar motion, and local air pressure changes. Finally, the residuals of the least-squares fit, the gravity time series and distribution histograms, as well as various operating parameters and environmental sensor readings are presented in graphical form.

The instrumental accuracy of the FG5 is about 1–2 µGal^18^; a precision of 1 µGal can be achieved within 1 hour at most sites, if the FG5 runs continuously. The instrumental uncertainty is 2.2 µGal^18^ and it results from several error sources such as magnetic field gradient, residual air pressure, electrostatics attraction of apparatus, differential temperature, air gap modulation, laser wavelength, comer-cube rotation, verticality, Coriolis effect, floor recoil and tilt, electronic phase shift, frequency standard, glass wedges and diffraction limit.

The Scintrex CG-6 Autograv relative gravimeter, managed by INGV-OE, is a land gravity meter with a renowned quartz sensor technology housed in a rugged, yet lightweight enclosure. This instrument features a worldwide measurement range (over 8000 mGal) and represents the state-of-the-art technology of spring-type gravimeters for high precision surveys. The manufacturer declares a reading resolution of 0.1 μGal and a repeatability of 5 μGal^19^. The CG-6 can operate under both indoor and outdoor conditions and, thanks to its limited size and weight (21.5 cm × 21 cm × 24 cm; weight of 5.5 kg), can be hand-transported and used for small-scale surveys in areas not accessible by car. The CG-6 is powered by 2 × 6.8 Ah (10.8 V) rechargeable lithium smart batteries, allowing a full day operation at 25 °C. The operating temperature range is from −40 °C to + 45 °C. The CG-6 Autograv operates by measuring the change in displacement of a proof mass suspended to a quartz spring. Displacements of the proof mass are sensed by a capacitive transducer, while an electrostatic feedback system keeps the mass in the null position by applying a DC voltage to the capacitor plates. The feedback voltage is proportional to the gravity change that caused the displacement of the proof mass and provides the output in terms of gravity readings.

The static instrumental drift rate of the CG-6, which is corrected by the real-time automatic compensator of the instrument, was investigated by repeating continuous measurement sessions in the gravity laboratory of INGV-OE. Using the standard drift calibration test^19^, the static drift rate was found to be about 0.050 mGal/day. Conversely, the dynamic drift rate, estimated when the instrument was moved from a station to the other, during surveys, was found to be on the order of a few μGal/day.

### Site positioning

In order to georeference the network benchmarks, for each site a mixed terrestrial satellite surveying campaign was conducted (Table 1).

**Table 1:** Reference epochs and final coordinates of the sites obtained after the joint adjustments of GNSS and topographic observations.

RETE H0						
G0 reference sites	Measurement epoch	Geodetic coordinates ITRF2014			GEOID ITALGEO	Orthometric heights
		h(m)	LAT	LONG	N(m)	H(m)
CATANIA	2022.419	71.32	37.5137922	15.0819436	41.11	30.21
CENTURIPE	2022.413	713.21	37.6271528	14.7378358	42.09	671.12
MILAZZO	2022.411	44.74	38.2208923	15.2420093	42.75	1.99
NOTO	2022.416	124.78	36.8760409	14.9890523	41.36	83.42
PALERMO	2023.015	87.80	38.1056028	13.3484906	43.52	44.28

To achieve the orthometric heights, useful to refer the gravity measurements to the equipotential surface, it is necessary to know the value of the geoidal undulation in the sites. This value can be retrieved by the regional model of Italian geoid estimated by the International Geoid Service at Politecnico di Milano^25^.

For Milazzo, Centuripe, Noto and Catania sites, two geodetic class GNSS receivers were used: A Geomax Zenith 35 Pro and an E-survey E300 Pro. These receivers are capable to acquire both code and phase observations on multiple frequencies on the signals transmitted by the four global systems (i.e. GPS, GLONASS, BEIDOU and GALILEO). The observations are stored in standard format files (RINEX) and processed in order to estimate absolute coordinates of outdoor reference points. Moreover, being NOTO one of the IERS reference stations, we also provide the geocentric Cartesian components of the baselines between the new gravimetric reference point and the reference benchmarks of VLBI and GNSS NOT1.

In all the sites, a Total Station was then used in order to measure angles and distances between the outdoor references to the indoor gravimetric points.

For Milazzo, Centuripe, Noto and Catania sites, the Total Station used is a Stonex R2Plus which, as reported by the manufacturer, can measure distances up to 3000 m using a prism and the accuracy corresponds to (2 + 2e^10-6*D) mm. The minimum angular reading corresponds to 0.0002 gon.

For Palermo site a Total Station used is a Leica TPS 1105 (Hexagon AB, Sweden) and topographic right-angle prisms were employed. The system is characterized by an angle measurement accuracy of 3” (1 mgon) and a distance measurement (Infrared sensor, IR) accuracy (ISO 17123-4) in standard mode of 2 mm. Only for this site a precise geometric leveling was carried out, with a digital level Leica DN03 (Hexagon AB, Sweden) with standard deviation height measurement per 1 km double-run (ISO 17123-2) with standard staffs 1.0 mm, resolution height measurement 0.01 mm, compensator setting accuracy 0.3”.

### Absolute gravity observations and data processing

In order to measure the absolute gravity, FG5#238 was positioned directly on the floor (usually tiles) of the chosen sites, once appropriate noise and stability checks were completed.

Absolute gravity measurements (sessions) generally consisted of 15/20 sets of 50/100 drops each; each set lasts 1 hour, implying a total measurement time of 15–20 hours. Even if the software supplied by Micro-g LaCoste provides the value of the local gravity in near real-time, more accurate analyses were performed in post-processing. Although environmental conditions were, in general, good, in some cases, due to local noise (e.g. the presence of the heavy spinning VLBI antenna at Noto station), increases were observed in the drop-to-drop scatter of the observations. In these cases, we reduced the set scatter by removing the anomalous values, thus achieving the desired precision. Thus, the processed sets usually mismatch the acquired sets.

The combined standard uncertainty of the calculated g values was calculated taking into account the contributions due to the instrumental uncertainty, the site-dependent uncertainty, the uncertainty related to the vertical gravity gradient determination and the statistical uncertainty due to the scattering. The statistical uncertainty (*δ*_*stat*_), is given by the standard deviations of the absolute gravity values obtained for each set (*σ*_*set*_) divided by the square root of the number of sets, *N*_*set*_:

1 $${\delta }_{stat}={\sigma }_{set}/\sqrt{{N}_{set}}$$

The combined uncertainty (*δ*_*abs*_) for the final gravity value is given by:

2 $${\delta }_{abs}=\sqrt{{\delta }_{stat}^{2}+{\delta }_{sys}^{2}}$$

where *δ*_*sys*_ is the systematic uncertainty that is due to different components of the measurements that can be grouped into separate areas: (i) modeling of geophysical processes (i.e. Barometric, Polar Motion, Earth Tide, Ocean Loading); (ii) system (Laser, Clock and System Model); (iii) environment (generally site dependent); (iv) set-up (depending on both the instrument and the operator) and (v) gradient (if applicable). For the FG5, we considered 3.14 µGal as the best representative value of the systematic uncertainty.

Although the g value at the absolute stations was measured at about 1.29 m from the ground (Z_actual height), using the Vertical Gravity Gradient (VGG) measured at each station, we calculated the g values at 0.72 m (the typical measuring height of the Micro-g LaCoste A10 absolute gravimeter) and at ground. The g at different heights was calculated by the following equation:

3 $${g}_{h1}=(-(h-{h}_{1})\ast {\rm{VGG}}+g)$$

where:

*g*_*h*1_ = absolute gravity value at desired height (*h*_1_)

*h* = height at which the g value was measured (Z_actual height)

VGG = vertical gravity gradient (measured with a relative gravimeter, as described in the following paragraph).

*g* = absolute gravity value measured at Z_actual height

The values of the absolute gravity with the respective combined uncertainties (*δ*_*abs*_) measured at the five Sicilian stations are reported in Tables 2, 4, 6, 8, 10.

**Table 2:** Absolute gravity measurement collected at Catania station (CTA) on 29 and 30 September 2022 by FG5#238 absolute gravimeter.

Absolute gravity measurements at CATANIA station (CTA)					
Date Time UTC [from ÷ to] P (hPa)	Meter and measured height (m)	Number of sets/drops per set/total drops	g at measured height (µGal)	g at 0.72 m (µGal)	g at ground (µGal)
29-30/09/2022	FG5#238	18/100/1800	980031286.1	980031446.9	980031649.5
10:47 ÷ 04:00	1.2917		±3.4	±3.4	±3.7
1009.63					
30/09/2022 - Vertical gravity gradient dg/dh = −281.4 ± 1.2 µGal/m					

From left to the right of the table are indicated: data, measurement time in UTC and the mean value of the nominal air pressure; the used instrument and the measured height h (elevation above the ground to which g is measured); the number of sets, drops per sets and total number of drops for each session; absolute gravity values at the measured height h; absolute gravity values at the transferred height of 0.72 m and at ground. The combined uncertainties related to the g values at different heights are also reported. On the bottom the vertical gravity gradient value measured on 30 September 2022 by the Scintrex CG-6 and related uncertainty is reported.

### Vertical gravity gradient measurements

The VGG values at CTA, CNT, MLZ, NTO, and UNPA absolute stations were measured using the Scintrex CG-6 relative gravity meter.

**Fig. 2:**
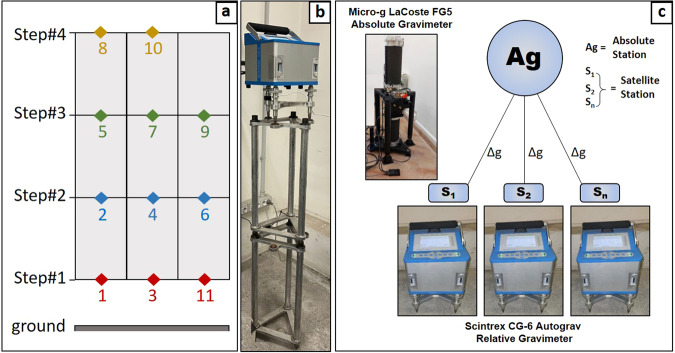
(**a**) procedure to measure the vertical gravity gradient indicating the measurement progression from 1 to 11 at different heights from the ground (Step#1 = about 15 cm; Step#2 = about 45 cm; Step#3 = about 70 cm; Step#4 = about 125 cm); (**b**) The Scintrex CG-6 relative gravimeter during vertical gravity gradient measurement; (**c**) scheme of the connection to calculate the gravity difference (Δg) between the indoor absolute point with one or more outdoor satellite station/s using the CG-6 Scintrex relative gravimeter.

The instrument was positioned at different heights from the ground, using a specifically designed perch consisting of assembled and overlapped modules of various heights (Fig. 2).

To define the VGG measurements with the CG-6 are taken at four different heights: step#1 = 15 cm; step#2 = 45 cm; step#3 = 70 cm; step#4 = 125 cm (Fig. 2a). These values also include the height of the tripod (about 15 cm) used for leveling the instrument, plus a fixed height of 2.591 cm, which corresponds to the vertical distance between the CG-6 sensor and the bottom of the instrument.

The measurements were executed using the step method; for each height the CG-6 recorded three values, each representing the average of 60 samples. All the adjacent elevations were connected at least three times. After the correction for the Earth tide, the VGG was obtained by fitting the following equation model to the experimental data, i.e. the g values, each recorded at a given acquisition time *t*:

4 $$g=\gamma \ast h+a\ast t+k$$

where the parameters *γ*, *h*, *a*, *k* are the vertical gravity gradient, the elevation from the floor, the instrumental drift, and the gravity offset, respectively.

The scheduled procedure for the VGG estimation with the measurement progression at different heights, as well as a picture showing the CG-6 during a measurement session are reported in Fig. 2a,b. Results obtained at each station are given in Tables 2, 4, 6, 8, 10.

### Measurements between absolute and satellite points

In order to “transport” the absolute g values outside the buildings hosting the absolute gravity stations, outdoor satellite gravimetric sites were also established; here the absolute value of g has been reported through relative measurements (Fig. 2c). The presence of the satellite gravimetric sites is important in order to perform rapid links during any relative gravimetric field survey that need to be linked to the absolute gravity value.

Using the Scintrex CG-6, we connected at least three times each satellite station with the nearby absolute-indoor site in order to determine high-precision Δgs and their uncertainties. These absolute values were transferred to 0 m (ground) through the VGG determined at each indoor absolute station. Gravity values at the satellite stations have been then determined by adding the difference (Δg) measured between the absolute station with the relative satellite stations at 0 m. The uncertainty of the calculated g value at the satellite stations was determined according to the equation:

5 $${\delta }_{sat}=\sqrt{{\delta }_{gabs}^{2}+{\delta }_{\Delta g}^{2}}$$

where *δ*_*gabs*_ is the combined uncertainty of g at absolute station at ground, and *δΔg* is the uncertainty associated to the measured gravity difference (Δg).

### Coordinate measurements

For each site a mixed terrestrial and satellite surveying campaign was conducted. For all sites, except Palermo, two outdoor reference points were established and surveyed by two geodetic class GNSS receivers with an acquisition rate of 1 Hz. The reference points were surveyed for about three hours leading to an observation dataset of at least 10 K epochs for each point.

The observations, stored in standard RINEX format files, were processed by the Precise Point Positioning (PPP) approach exploiting the availability of precise products (precise satellite orbits, clocks etc). PPP estimations were performed using a web service made available by the Canadian Geodetic Survey^20^ obtaining, for all the outdoors reference points, the coordinates in ITRF2014^21^ with an average accuracy of a few centimeters.

Then, a Total Station was used to link the outdoor reference points to the indoor gravimetric points. A local Cartesian coordinate system^22^ (East, North and Up components) has been defined with origin in the mean point between the two outdoor reference points, and the three-dimensional observations collected by the Total Station (i.e. zenithal angles, azimuthal directions and three-dimensional distances) have been used. All the resulting observation equations have been linearized with respect to approximate values and a weighted least square adjustment (with respect points coordinates) iterative process was carried out. In the adjustment, the outdoor reference points coordinates were constrained to the PPP estimated with their variances (provided by the PPP estimation), and the instrumental precision of the Total Station observations has been considered for the weights model, leading to the estimation for all network points of their three-dimensional local Cartesian coordinates (E, N, Up). The gravimetric point coordinates resulting from the adjustment have been finally transformed from the local Cartesian system to geodetic coordinates (i.e. Latitude, Longitude and ellipsoidal height).

For Palermo site, a similar approach was carried out, adding a precise geometric levelling. For Catania, Noto and Palermo, given the presence of a co-located GNSS permanent station, the network has been extended in order to include the permanent stations and improve the redundancy of the surveys.

For each site, the description of the surveying network, the results of the PPP estimation of the outdoor reference points and the results of the network adjustment are described in the following subsections.

## Data Records

Data records are available at the Zenodo Repository^23^, through the https://zenodo.org/record/8365542.

The database includes subfolders for each of the five measured gravity absolute stations, named: Palermo (UNPA), Noto (NTO), Milazzo (MLZ), Centuripe (CNT), Catania (CTA). Besides the raw data (drops; gfs format) averaged to obtain the final g value, each subfolder also contains information about the measurements session and instrument characteristics. Further, a txt file summarizes information such as: instrument data, station data, processing results, acquisition settings, gravity corrections, data uncertainties.

### Catania station (CTA)

#### Monograph

Figure A1 (supplementary information) shows the monograph and the pictures of the absolute gravity and height station in Catania. The reference station of Catania (CTA; see supplementary information) is located at the Gravity Laboratory of INGV-OE. When measuring the absolute gravimeter is placed on a concrete pillar insulated from the building. The laboratory conditions are optimal, with low humidity and stable ambient temperature. The CTA station is intended as a primary reference station for the gravity monitoring network of Mt. Etna volcano. Here, since 2009, FG5#238 gravimeter is maintained and tested and absolute gravity measurements are regularly carried out^8–10^.

#### Gravity observations

FG5#238 measured from 29 to 30 September 2022, and 18 measurement sets, each consisting of 100 drops, were acquired in about 18 hours (mostly during the night). The environmental parameters during the measurement sessions were very stable. Results of absolute measurements, as well as the VGG value measured on 30 September 2022, are reported in Table 2.

#### GNSS and height determination at Catania station

The measurements were carried out on June 2, 2022 by surveying 2 GNSS sites located in the courtyard of the INGV-OE building (200 and 300, see supplementary information) and subsequently measuring the distances and angles between these sites and CTA gravity station with the total station (100). The survey has also been connected to the permanent GNSS stations of EIIV operating on the rooftop terrace. The results after the least squares adjustment are reported in Table 3.

**Table 3:** Upper panel: Coordinates and rms of the GNSS sites after the PPP solution; Mid panel: Coordinates and rms of the whole network in a local Cartesian frame; Lower panel: Epoch of measurement and final geodetic coordinates of the absolute site.

Precise Point Positioning coordinates and accuracies of the outdoor reference points.						
Name	LAT (deg)	σ LAT (m)	LON (deg)	σ LON (m)	h (m)	σ h (m)
200	37.5140672	0.009	15.0817294	0.013	75.94	0.05
300	37.5136957	0.011	15.0820463	0.035	84.29	0.04
EIIV	37.5136048	0.002	15.0820864	0.002	88.89	0.01
**Local cartesian coordinates of the network resulting from the adjustment**						
**Name**	**E** (**m**)	**σ E** (**m**)	**N** (**m**)	**σ N** (**m**)	**h** (**m**)	**h σ** (**m**)
100	−4.114	0.004	−16.565	0.006	71.74	0.04
200	−14.004	<0.001	20.612	<0.001	75.94	0.05
300	13.979	<0.001	−20.611	<0.001	84.29	0.04
EIIV	17.558	<0.001	−30.703	<0.001	88.89	0.01
CTA	4.929	0.004	−9.902	0.006	71.32	0.03
**Geodetic coordinates of the CATANIA gravimetric point** (**in ITRF2014**)						
DOY	153/2022					
Name	LAT (deg)	LON (deg)	h (m)			
CTA	37.5137922	15.0819436	71.32			

### Centuripe station (CNT)

#### Monograph

Centuripe absolute station (CNT) was installed and measured for the first time in 1991 in the frame of the absolute gravity and gradiometry measurements carried out on the active volcanoes of Southern Italy^8^. At that time, measurements at this absolute station were performed using the IMGC absolute gravimeter developed by Istituto Nazionale di Ricerca Metrologica (INRiM, Torino).

The monograph and pictures of the reference station CNT are showed in the supplementary information (Fig. A2). The station was set up within the school “G. Verga”, located at the top of the Centuripe village (see supplementary information). Specifically, the gravity station was settled in the archive room situated in the underground floor, in the same place where absolute gravity measurements were carried out in the 90 s. Two satellite stations were also measured. Due to the renovation and variations of the building hosting the school during years, measurements at the satellite station on the threshold of the principal gate of the school in June 2022 were not performed in the same place where measurements were made in the 1990s^8^. A new satellite station (CNT_S1) was located in the new threshold, on the left side of the principal entry (see supplementary information). As for the satellite station CNT_S2 (on the marble threshold at the right side entry of the Chiesa Madre), the 2022 measurements were performed at the same point measured by Berrino^8^.

#### Gravity observations

The absolute gravity station Centuripe was measured during May 31 to June 01 2022. 19 measurement sets, each consisting of 100 drops (in total 1900 drops), were acquired in about 19 hours (mostly during the night). Table 4 reports all the information about the gravity measurements, as well as the values of g at measured and transferred heights. The associated combined uncertainties are also reported. At the bottom of the table the value of the local VGG, measured on 23 December 2021, and the measured Δgs between CNT and the satellite gravity stations CNT_S1 and CNT_S2, with their uncertainties, are also reported.

**Table 4:** Absolute gravity measurement collected at Centuripe station (CNT) on 31 May and 01 June 2022 by FG5#238 absolute gravimeter.

Absolute gravity measurements at CENTURIPE station (CNT)					
Date Time UTC [from ÷ to] P (hPa)	Meter and measured height (m)	Number of sets/drops per set/total drops	g at measured height (µGal)	g at 0.72 m (µGal)	g at ground (µGal)
31/05-01/06/2022	FG5#238	19/100/1900	979822689.3	979822900.8	979823166.6
12:49 ÷ 06:52	1.2927		±3.4	±3.4	±3.4
935.18					
23/12/2021 - Vertical gravity gradient dg/dh = −369.2 ± 0.3 µGal/m					
23/12/2021 - Δg at the external relative satellite station (CNT_S1) with respect to the absolute site at ground = −985 ± 6.8 µGal; g = 979822181.6 ± 7.6 µGal					
23/12/2021 - Δg at the external relative satellite station (CNT_S2) with respect to the absolute site at ground = −11000 ± 3.8 µGal; g = 979812166.6 ± 5.1 µGal.					

In the table, from left to the right are reported: data, measurement time in UTC and the mean value of the nominal air pressure; the used instrument and the measured height h (elevation above the ground to which g is measured); the number of sets, drops per sets and total number of drops for each session; absolute gravity values at the measured height h; absolute gravity values at the transferred height of 0.72 m and at ground. The combined uncertainties related to the g values at different heights are also reported. On the bottom the vertical gravity gradient value and the Δg measured at the external satellite stations (CNT_S1 and CNT_S2) on 23 December 2021 by the Scintrex CG-6 and related uncertainties are presented.

#### Coordinates determination

The measurements were carried out on May 31, 2022 by surveying 2 GNSS sites located in the courtyard of the school (200 and 300, see supplementary information) and subsequently measuring the distances and angles between these sites and the gravimetric site CNT with the total station (100). The results after the least squares adjustment are reported in Table 5.

**Table 5:** Upper panel: Coordinates and rms of the GNSS sites after the PPP solution; Mid panel: Coordinates and rms of the whole network in a local Cartesian frame; Lower panel: Epoch of measurement and final geodetic coordinates of the absolute site.

Precise Point Positioning coordinates and accuracies of the outdoor reference points						
Name	LAT (deg)	σ LAT (m)	LON (deg)	σ LON (m)	h (m)	σ h (m)
200	37.6274036	0.008	14.7377000	0.015	716.25	0.04
300	37.6268208	0.013	14.7379785	0.010	713.30	0.07
**Local cartesian coordinates of the network resulting from the adjustment**						
**Name**	**E** (**m**)	**σ E** (**m**)	**N** (**m**)	**σ N** (**m**)	**h** (**m**)	**σ h** (**m**)
100	−5.981	0.001	3.465	0.001	713.02	0.03
200	−12.292	0.002	32.345	0.002	716.25	0.04
300	12.292	0.002	−32.345	0.002	713.30	0.07
CNT	−0.307	0.002	4.502	0.001	713.21	0.03
**Geodetic coordinates of the Centuripe gravimetric point** (**in ITRF2014**)						
DOY	151/2022					
**Name**	**LAT (deg)**	**LON (deg)**	**h (m)**			
CNT	37.6271528	14.7378358	713.21			

### Milazzo station (MLZ)

#### Monograph

The monograph and pictures of the absolute gravity station MLZ are provided in the supplementary information (Fig. A3). The station was set up within the Town Hall of Milazzo, in the same place where absolute gravity and vertical gravity gradient measurements were made in the 90 s. The satellite station (MLZ_S1) was placed on the marble threshold on the left side of one of the entries of the same building^24^ (see supplementary information).

#### Gravity observations

On 30 and 31 May 2022, the FG5#238 was installed at MLZ and a measurement session was performed during about 17 hours (mostly during the night), consisting of 17 sets (100 drops each). At the end of the measurement session, the VGG was measured. Furthermore, measurements at one outdoor satellite station were also carried out to evaluate the gravity difference (Δg) with respect to the indoor absolute point. Table 6 shows the results of the measurements performed in 2022.

**Table 6:** Absolute gravity measurement collected at Milazzo station (MLZ) on 30 and 31 May 2022 by FG5#238 absolute gravimeter.

Absolute gravity measurements at MILAZZO station (MLZ)					
Date Time UTC [from ÷ to] P (hPa)	Meter and measured height (m)	Number of sets/drops per set/total drops	g at measured height (µGal)	g at 0.72 m (µGal)	g at ground (µGal)
30-31/05/2022	FG5#238	17/100/1700	980103887.3	980104061.9	980104280.7
14:49 ÷ 06:15	1.2942		±3.3	±3.4	±3.4
1013.01					
31/05/2022 - Vertical gravity gradient dg/dh = −304 ± 0.4 µGal/m					
31/05/2022 - Δg at the external relative satellite station with respect to the absolute site at ground = −82.4 ± 4.4 µGal g = 980104198.3 ± 5.5 µGal					

In the table, from left to the right are reported: data, measurement time in UTC and the mean value of the nominal air pressure; the used instrument and the measured height h (elevation above the ground to which g is measured); the number of sets, drops per sets and total number of drops for each session; absolute gravity values at the measured height h; absolute gravity values at the transferred height of 0.72 m and at ground. The combined uncertainties related to the g values at different heights are also reported. On the bottom the vertical gravity gradient value and the Δg measured at the external satellite station (MLZ_S1) on 31 May 2022 by the Scintrex CG-6 and related uncertainties are presented.

#### Coordinates determination

The measurements were carried out on May 30, 2022 by surveying 2 GNSS sites located in Caio Duilio square of Milazzo (200 and 300, see supplementary information) and subsequently measuring the distances and angles between these sites and the gravimetric site MLZ with the total station (100), which was located outdoor just in front of the room hosting the gravimetric site. The results after the least squares adjustment are reported in Table 7.

**Table 7:** Upper panel: Coordinates and rms of the GNSS sites after the PPP solution; Mid panel: Coordinates and rms of the whole network in a local Cartesian frame; Lower panel: Epoch of measurement and final geodetic coordinates of the absolute site.

Precise Point Positioning coordinates and accuracies of the outdoor reference points						
Name	LAT (deg)	σ LAT (m)	LON (deg)	σ LON (m)	h (m)	σ h (m)
200	38.2208174	0.009	15.2414792	0.011	44.82	0.04
300	38.2205460	0.013	15.2414053	0.033	44.38	0.05
**Local cartesian coordinates of the network resulting from the adjustment**						
**Name**	**E** (**m**)	**σ E** (**m**)	**N** (**m**)	**σ N** (**m**)	**h** (**m**)	**σ h** (**m**)
100	39.550	0.034	31.886	0.046	44.38	0.01
200	3.245	0.013	15.058	0.015	44.82	0.04
300	−3.245	0.013	−15.058	0.015	44.38	0.05
MLZ	49.650	0.027	23.376	0.054	44.74	0.01
**Geodetic coordinates of the Milazzo gravimetric point** (**in ITRF2014**)						
DOY	150/2022					
**Name**	**LAT (deg)**	**LON (deg)**	**h (m)**			
MLZ	38.2208923	15.2420093	44.74			

### Noto station (NTO)

#### Monograph

The monograph and pictures of the absolute gravity station in Noto are showed in the supplementary information (Fig. A4). The NTO station was set in the basement of the radio telescope (VLBI antenna) managed by Istituto Nazionale di Astrofisica, located at close distance from the town of Noto (Province of Siracusa). This is the same place where absolute gravity and VGG measurements were made in the 90s^25^. The satellite station was located on a wall in front of the gate of the external main entry.

#### Gravity observations

The absolute g value at NTO station was measured during 23 to 24 March 2022. In about 19 hours, 1150 drops were collected, the majority during the night. The VGG and the gravity difference between NTO station and an external satellite station were also measured. Due to the presence of the heavy spinning VLBI antenna, the drop-to-drop scatter of the observations was very high during the movement of the antenna. For this reason, we decided to reduce the number of drops for each set from 100 to 50. Furthermore, during the post-processing of the data, we removed some outlier values, in order to obtain the desired precision. Thus, the number of processed sets is less than the acquired ones.

The gravity values at different heights, as well as the Δg measured between NTO and the external satellite station (NTO_S1) are reported in Table 8.

**Table 8:** Absolute gravity measurement collected at Noto station (NTO) on 23 and 24 March 2022 by FG5#238 absolute gravimeter.

Absolute gravity measurements at NOTO station (NTO)					
Date Time UTC [from ÷ to] P (hPa)	Meter and measured height (m)	Number of sets/drops per set/total drops	g at measured height (µGal)	g at 0.72 m (µGal)	g at ground (µGal)
23-24/03/2022	FG5#238	23/50/1150	979992566.8	979992745.2	979992969.2
12:01 ÷ 07:05	1.2932		±3.5	±3.5	±3.5
1003.27					
14/12/2021 - Vertical gravity gradient dg/dh = −311.1 ± 0.5 µGal/m					
14/12/2021 - Δg at the external relative satellite station with respect to the absolute site at ground = −394 ± 5 µGal; g = 979992575.2 ± 6.1 µGal					

In the table, from left to the right are reported: data, measurement time in UTC and the mean value of the nominal air pressure; the used instrument and the measured height h (elevation above the ground to which g is measured); the number of sets, drops per sets and total number of drops for each session; absolute gravity values at the measured height h; absolute gravity values at the transferred height of 0.72 m and at ground. The combined uncertainties related to the g values at different heights are also reported. On the bottom the value of the vertical gravity gradient and the Δg measured at the external satellite station (NTO_S1) on 14 December 2021 by the Scintrex CG-6 and related uncertainties are presented.

#### Coordinates determination

The measurements were carried out on June 1, 2022 by surveying 2 GNSS sites located in the courtyard of the CNR area (200 and 300, see supplementary information) and subsequently measuring the distances and angles between these sites and a site (100) located just outdoor the reinforced concrete basis of the VLBI antenna, hosting the NTO absolute gravity station. The results, after the least squares adjustment, are reported in Table 9a.

**Table 9:** (**a**) Upper panel: Coordinates and rms of the GNSS sites after the PPP solution; Mid panel: Coordinates and rms of the whole network in a local Cartesian frame; Lower panel: Epoch of measurement and final geodetic coordinates of the absolute site. (**b**) Geocentric cartesian coordinates of NOTO VLBI, Gravimetric and GNSS reference points. Components of the geocentric cartesian and local cartesian system baselines between NTO and the other two reference points.

(a)						
Precise Point Positioning coordinates and accuracies of the outdoor reference points						
Name	LAT (deg)	σ LAT (m)	LON (deg)	σ LON (m)	h (m)	σ h (m)
200	36.8765459	0.005	14.9885057	0.006	125.42	0.02
300	36.8760949	0.008	14.9897969	0.021	125.31	0.03
NOT1	36.8758475	0.003	14.9897915	0.003	126.32	0.01
**Local cartesian coordinates of the network resulting from the adjustment**						
**Name**	**E (m)**	**σ E (m)**	**N (m)**	**σ N (m)**	**h (m)**	**σ h (m)**
100	−4.789	0.003	−16.262	0.006	125.442	0.02
200	−57.559	0.005	25.026	0.006	125.42	0.02
300	57.541	0.003	−25.019	0.009	125.31	0.03
NOT1	57.073	0.003	−52.483	0.003	126.32	0.01
NTO	−8.826	0.003	−31.013	0.007	124.78	0.02
**Geodetic coordinates of the NOTO gravimetric point (in ITRF2014)**						
DOY	152/2022					
**Name**	**LAT**	**LON**	**h (m)**			
NTO	36.8760409	14.9890523	124.78			

(b)						
ITRF2014 Epoch 2022.5	X (m)	Y (m)	Z (m)			
VLBI (V)	4934562.664	1321201.706	3806484.878			
NTO (G)	4934549.328	1321198.007	3806472.632			
NOT1 (N)	4934545.914	1321265.309	3806456.387			
	**Delta X (m)**	**Delta Y (m)**	**Delta (Z) (m)**	**3D distance (m)**		
Baseline (G-V)	−13.336	−3.699	−12.246	18.479		
Baseline (G-N)	3.414	−67.302	16.245	69.319		
	**Delta E (m)**	**Delta N (m)**	**Delta Up (m)**	**2D distance (m)**		
Local Baseline (G-V)	−0.124	−1.491	−18.419	1.497		
Local Baseline (G-N)	−65.895	21.461	−1.538	69.302		

Thanks to the topographic measurements, it is possible to indirectly connect the gravimetric point to the geodetic reference points of the Noto area (Table 9b).

In particular, from the ITRF2014 solution of IERS the IVS and GNSS rinex file were used to obtain the epoch 2010.0 cartesian coordinates of NOTO VLBI and GNSS (named NOT1) stations and their velocities. (https://itrf.ign.fr/en/solutions/itrf2014).

The coordinates have been propagated to surveys epoch (2022.5) through the provided velocities, then the geocentric Cartesian components of the baselines between the gravimetric reference point and the two other reference points were obtained, leading to tridimensional distances of 18.479 m (w.r.t. VLBI) and 69.319 m (w.r.t. NOT1). In order to appreciate the choice of the location of the gravimetric point, the East, North and Up components of the same baselines with respect to the local cartesian system with origin in VLBI reference point have been computed. It is possible to highlight that the horizontal distance between the gravimetric point NTO and VLBI is lower than 1.5 meters while the height difference is 18.419 m. On the other hand, the horizontal distance between NTO and NOT1 is 69.319 m while the height difference is around 1.5 meters.

### Palermo station (UNPA)

#### Monograph

The monograph and pictures of the absolute gravity station UNPA, located in Palermo, are reported in the supplementary information (Fig. A5). The station was set up at the University of Palermo, Engineering Department, inside the computer science room. The satellite station (UNPA_S1) was located on the marble threshold on the left side of the faculty main entry.

#### Gravity observations

The absolute gravity measurement at UNPA station was performed during 06 to 07 April 2022. The measurement session, consisting of 20 sets (50 drops each), was collected in about 17 hours (mostly during the night). The g value at different eighths, the value of the VGG, as well as the Δg between UNPA and UNPA_S1 are reported in Table 10.

**Table 10:** Absolute gravity measurement collected at Palermo station (UNPA) on 06 and 07 April 2022 by FG5#238 absolute gravimeter.

Absolute gravity measurements at PALERMO station (UNPA)					
Date Time UTC [from ÷ to] P (hPa)	Meter and measured height (m)	Number of sets/drops per set/total drops	g at measured height (µGal)	g at 0.72 m (µGal)	g at ground (µGal)
06-07/04/2022	FG5#238	20/50/1000	980035246.2	980035408.8	980035615.9
13:02 ÷ 05:42	1.2852		±3.6	±3.6	±3.6
1007.5					
15/12/2021 - Vertical gravity gradient dg/dh = −287.7 ± 0.3 µGal/m					
15/12/2021 - Δg at the external relative satellite station (UNPA_S1) with respect to the absolute site at ground = −12 ± 7 µGal; g = 980035603.9 ± 7.9 µGal					

In the table, from left to the right are reported: data, measurement time in UTC and the mean value of the nominal air pressure; the used instrument and the measured height h (elevation above the ground to which g is measured); the number of sets, drops per sets and total number of drops for each session; absolute gravity values at the measured height h; absolute gravity values at the transferred height of 0.72 m and at ground. The combined uncertainties related to the g values at different heights are also reported. On the bottom the value of the vertical gravity gradient and the Δg measured at the external satellite station (UNPA_S1) on 15 December 2021 by the Scintrex CG-6 and related uncertainties are presented.

#### Coordinates determination

In order to calculate the precise height of the UNPA gravimetric station, three types of topographical measurements were used: static GNSS survey, tacheometric leveling and precise geometric leveling. The planimetric measurements were carried out with a compound traverse on 15/01/2023.

During the first phase of the work, a benchmark was materialized in the neighborhood of the area in front of the gravimetric station, the coordinates of which were determined from the permanent GNSS PAUN station (inserted in the RDN network by IGMI), with a survey of 60′, cut off 15° and rate 10″. Once the coordinates of the benchmark were determined, an initial tacheometric leveling was carried out with total stationing, with respect to the ARP centre of the antenna, and subsequently, knowing the orthometric height of the point, a second precise geometric leveling was carried out, with a digital level. After, planimetric measurements of the gravimetric station were carried out with a compound traverse. The results after the least squares adjustment are reported in Table 11.

**Table 11:** Upper panel: Coordinates and rms of the GNSS sites after the solution; Lower panel: Epoch of measurement and final geodetic coordinates of the absolute site.

Coordinates and accuracies of the outdoor reference points						
Name	LAT (deg)	σ LAT (m)	LON (deg)	σ LON (m)	h (m)	σ h (m)
100	38.105047	0.004	13.348257	0.005	87.79	0.01
**Geodetic coordinates of the UPA gravimetric point** (**in ITRF2014**)						
DOY	015/2023					
**Name**	**LAT**	**LON**	**h (m)**			
UPA	38.1056028	13.3484906	87.804			

## Technical Validation

### Evaluation of the long-term behavior of the network stations

As stated in the previous sections, in choosing the sites forming the new networks, care was taken to select points where absolute gravity measurements were carried out in the past and/or where GPS/GNSS stations have been recording for several years. This strategy allowed us to evaluate the long-term behavior, in terms of stability, of the selected stations for the reference network.

#### Gravity: long-term evaluation

**Fig. 3:**
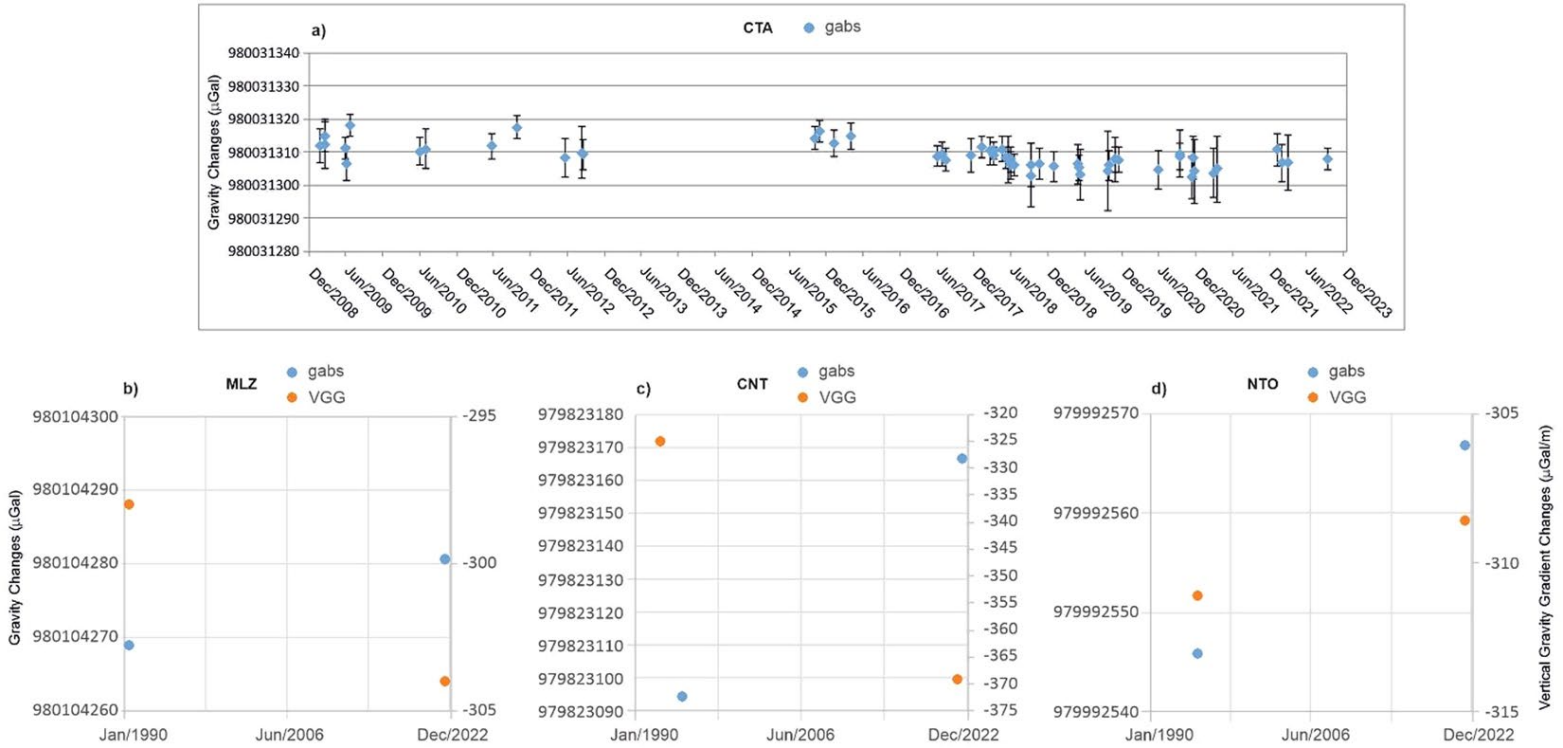
(**a**) Absolute gravity values at CTA (Catania) station during 2009–2022 period. Data are collected with the Micro-g LaCoste FG5#238 absolute gravimeter. The values of g are referred to an elevation of 1.2169 m from the ground, corresponding to the distance between the ground and the effective position of free-fall for FG5#238. The error bars represent the combined uncertainty. (**b**) Absolute gravity values at MLZ (Milazzo) station during 1990–2022 period. (**c**) Absolute gravity values at CNT (Centuripe) station during 1990–2022 period. (**d**) Absolute gravity values at NTO (Noto) station during 1990–2022 period.

For the Catania, Centuripe, Noto and Milazzo absolute gravity stations, an attempt to evaluate long-time gravity pattern change was made (Fig. 3), even if, with the exception of Catania, different gravimeters were used.

For the sake of homogeneity with previously collected data, we reanalyzed the values collected with the IMGC at Milazzo and Centuripe stations in the ‘90, taking into account the effect of the atmospheric pressure, not considered in the previous analyses.

Catania (CTA) - The value of g and the VGG value measured in September 2022 (Table 2), compared with the long data set existing for this station (about 35 measurements reported at the same height from the ground), confirms the long-term stability of this point (Fig. 3a). The averaged g value is 980031308.7 ± 5.2 µGal (combined uncertainty), with a standard deviation of 5.2 µGal calculated on 56 measurements.

Milazzo (MLZ) - The g value at ground measured in 2022 (Table 6), compared to the value measured on 26–28 June 1990 using the IMGC absolute gravimeter (g at measured height 0.920 m 980103994.6 ± 2 µGal; g at ground 980104268.8 ± 3 µGal^8,24^; reanalyzed), shows a slight variation of +11.9 ± 4.5 µGal (Fig. 3b). Comparison between the VGG measured in 2022 and that measured on 26–28 June 1990 returns a difference of −6 µGal/m. As for the satellite station (g at ground 980104202.8 ± 8 µGal), a difference of −4.5 ± 10 µGal was found in the period 1990–2022.

Centuripe (CNT) - The difference between the gravity value measured in 2022, referred at ground using the measured VGG (Table 4), and the value measured on 14–15 September 1994 using the IMGC absolute gravimeter (g at measured height 0.9494 m 979822788.9 ± 3 µGal; g at ground 979823094.7 ± 2 µGal^24^; reanalyzed) is +71.9 ± 4 µGal (Fig. 3c). The VGG measured in 2021 (Table 4) highlighted a variation of about −44 µGal/m with respect to the value observed in June 1992 (−325 ± 2 µGal/m) by Berrino^8^. The Δg measured in June 1992 between the external relative satellite station (CNT_S2) and the absolute site at ground was −10919.5 ± 5 µGal; g 979812175.2 ± 3 µGal. Thus, the variation of the absolute gravity value at CNT_S2 external point in the period 1992 - 2022 is −8.6 ± 6 µGal.

Noto (NTO) - The gravity value measured in 2022 has been compared with the value measured on 07 October 1994 using the IMGC absolute gravimeter (g at measured height 0.948 m 979992652.3 ± 3 µGal^23^). Since the local VGG was not measured in 1994, in order to make a comparison with the 2022 data, the 1994 value was reported at the measurement height of the FG5#238 (dh 0.345 m) using the theoretical VGG (−308.55 µGal/m). The g value referred at the FG5#238 measured height results 979992545.8 ± 3 µGal; thus, the change over the 1994–2022 time interval is +21 ± 5 µGal (Fig. 3d). No comparison at the satellite station has been possible as no links between the absolute point and any external point were established in the ‘90.

#### GPS/GNSS: long term evaluation

The Bernese Analysis Center of INGV analyzes GNSS observations of more than 1000 sites located in the Italian peninsula and surrounding regions by the Bernese software Ver. 5.0^26^, following the procedure described in Devoti *et al*.^27^. From the daily coordinate solutions, velocities at GPS stations are estimated by a linear weighted least square fit of all the coordinate time series simultaneously, using the full daily covariance matrices and modeling secular drifts, episodic offsets and annual sinusoids. The rms of the estimated velocities are in addition re-weighted taking into account the coordinate dispersion with respect to the linear model.

**Fig. 4:**
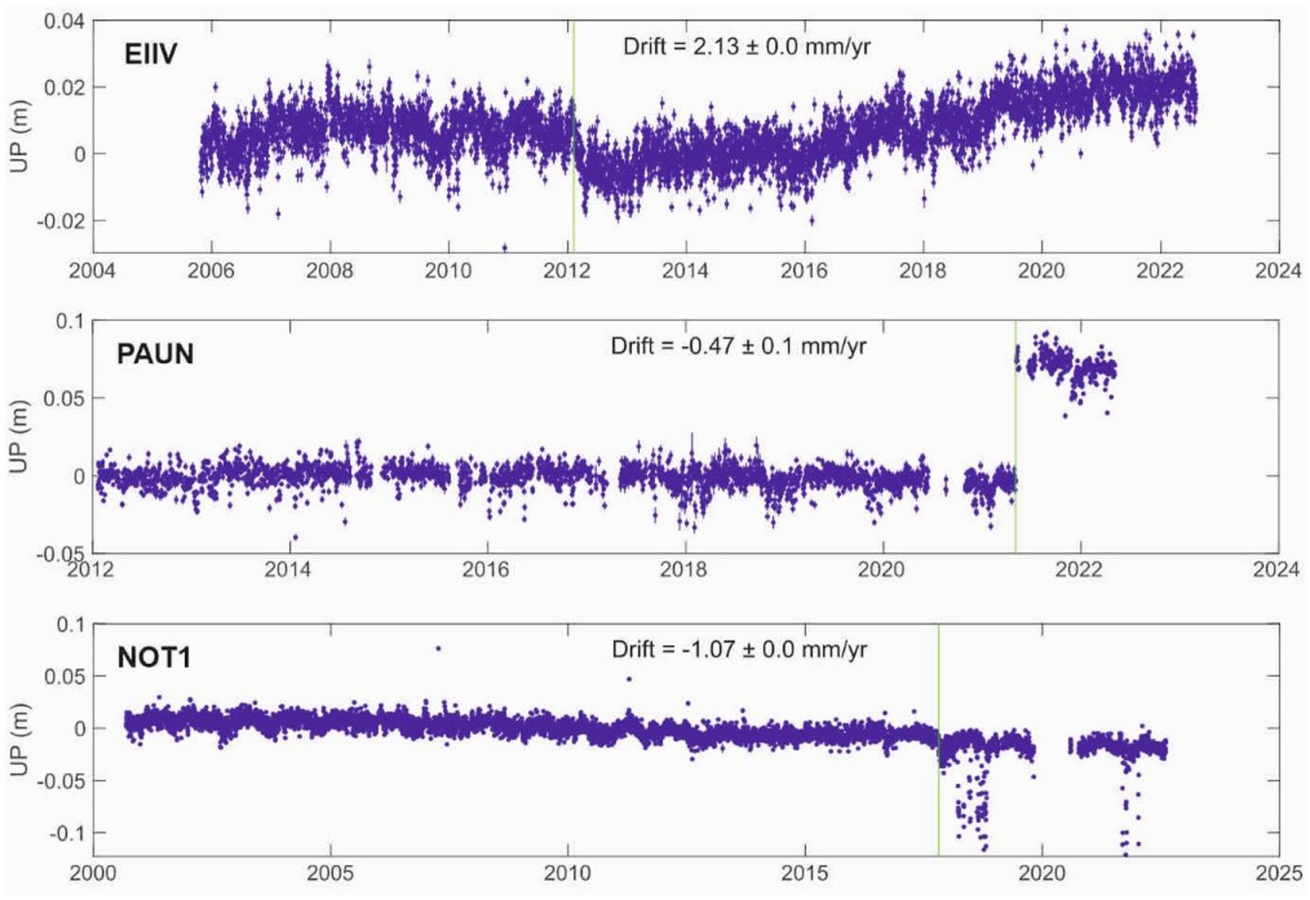
Time series of the Up component in ITRF2014 at EIIV, PAUN and NOT1 stations. Blue dots are the daily coordinate solutions, the green lines identify steps due to instrumental changes.

We have estimated the vertical velocities of the nearest GNSS permanent station co-located with the gravimetric sites of our network (Fig. 4). This condition is satisfied for Catania, Noto and Palermo for which we dispose of EIIV, NOT1 and PAUN GNSS permanent stations, respectively (Table 12). In the remaining two sites, Centuripe and Milazzo, we have obtained the vertical velocities of the nearest Permanent Scatterers from the European Ground Motion Service under the Copernicus Programme (https://land.copernicus.eu/pan-european/european-ground-motion-service).

**Table 12:** Vertical velocities, rms and time span of observations useful to estimate velocities.

Vertical velocities					
GNSS	Lon deg	Lat deg	Vu ± rmsVu mm/yr	dT yr	epoch
EIIV	15.0821	37.5136	+2.1 ± 1.6	16.8	Oct 2005-Aug 2022
NOT1	14.9898	36.8758	−1.1 ± 0.5	21.9	Sept 2000-Aug 2022
PAUN	13.3485	38.1055	−0.5 ± 2.0	10.3	Jan 2012-May 2022
**PS**(**InSAR**)					
Centuripe	14.7378	37.6271	+0.2 ± 1.5	5	Jan 2016-Dec 2021
Milazzo	15.2420	38.2209	−0.9 ± 1.0	5	Jan 2016-Dec 2021

The vertical velocities of PAUN, Centuripe and Milazzo are well within the estimated rms, NOT1 and EIIV are slightly larger than the rms (Fig. 4).

### Supplementary information

Supplementary Information

## Data Availability

No custom code has been used. Data processing has been described through the Methods section.
