# Supplementary material for: The first absolute gravity and height reference network in Sicily
Source: Sci Data. 2024 Apr 8;11:357. doi: 10.1038/s41597-024-03177-4 (PMC11001936; doi:10.1038/s41597-024-03177-4)
Supplement: Supplementary file 1 — Supplementary Information [file 41597_2024_3177_MOESM1_ESM.pdf]

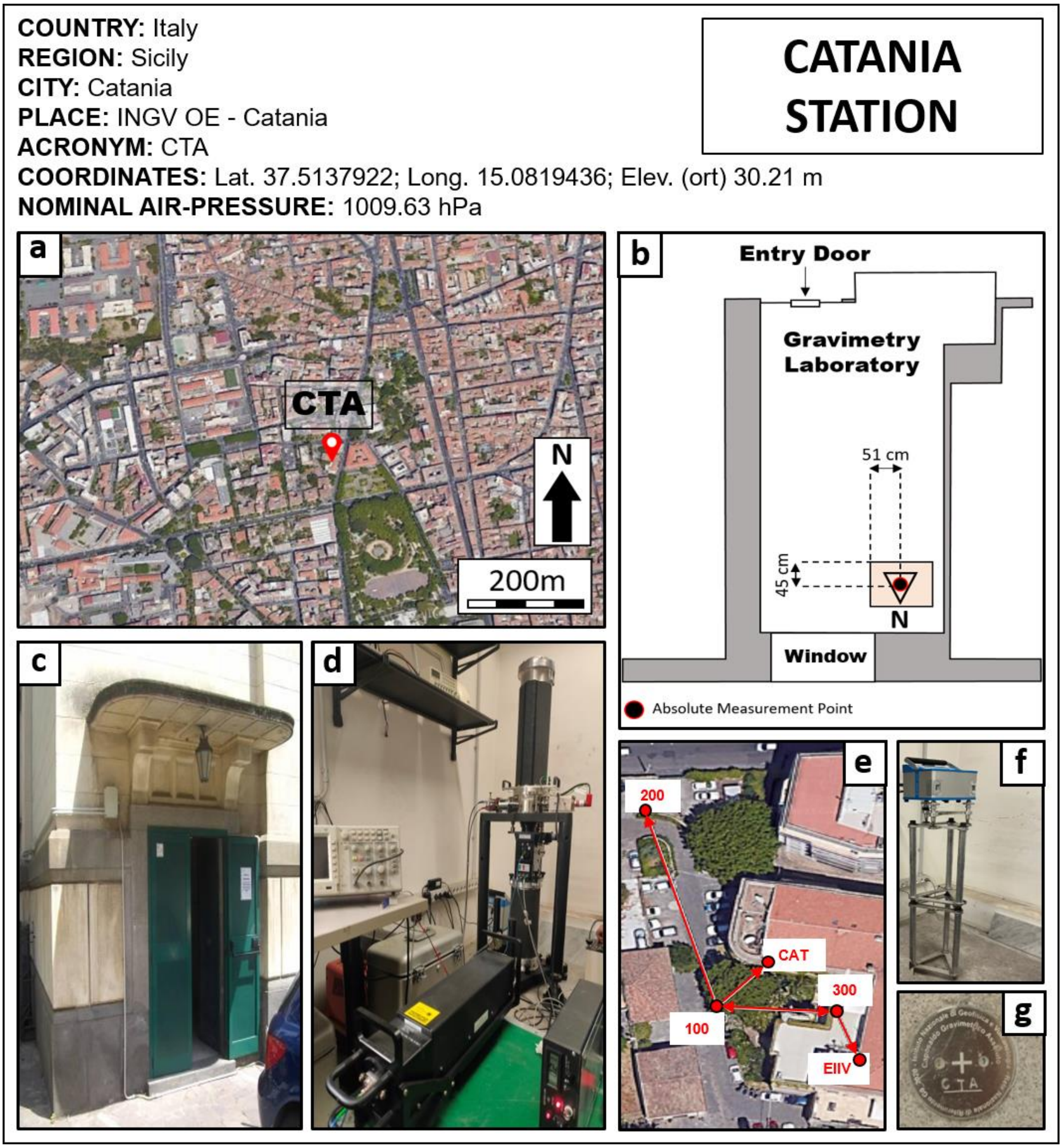


**Fig. A1** Schematic monograph and pictures of the absolute gravity station CTA. (**a)** Google Earth view of the area where the Catania absolute station is located (coordinates are described at the top of the figure); (**b)** detailed planimetry of the room with the exact location of the absolute measurement point; (**c)** external view of the building hosting the absolute gravity station; (**d)** the Micro-g LaCoste FG5#238 during the measurement session; (**e)** Catania surveyed network (CTA - absolute gravity point, 100 - Total Station, 200 and 300 - outdoor reference points (GNSS), EIIV - GNSS Permanent Station); (**f)** the Scintrex CG-6 relative gravimeter during vertical gravity gradient measurement; (**g)** photo of the stainless steel marker on which the acronym of the station is marked

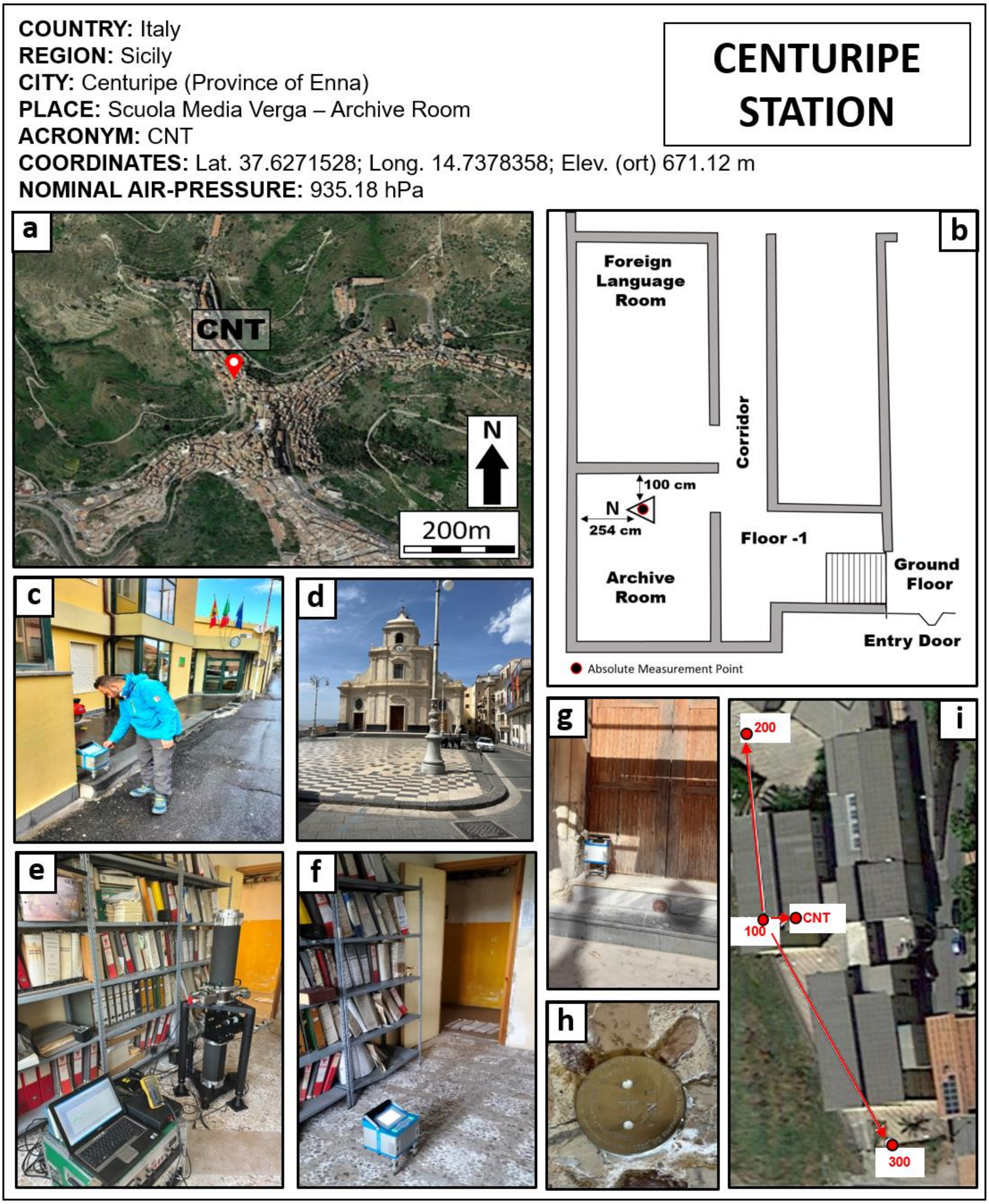


**Fig. A2** Monograph and pictures of the absolute gravity station CNT. (**a**) Google Earth view of the area where the Centuripe absolute station is located (coordinates are described at the top of the figure); (**b**) detailed planimetry of the room with the exact location and orientation of the absolute measurement point; (**c**) external view of the building hosting the absolute station and the external relative satellite station (CNT_S1) used for fast link with the absolute point; (**d**) external view of the relative satellite station (CNT_S2); (**e**) the Micro-g LaCoste FG5#238 during the measurement session; (**f**) the Scintrex CG-6 relative gravimeter during the vertical gravity gradient measurement; (**g**) Measurement of g at the relative satellite station (CNT_S2); (**h**) photo of the stainless steel marker on which the acronym of the station is marked; (**i**) Centuripe surveyed network (CNT - absolute gravity point, 100 - Total Station, 200 and 300 - outdoor reference points (GNSS))

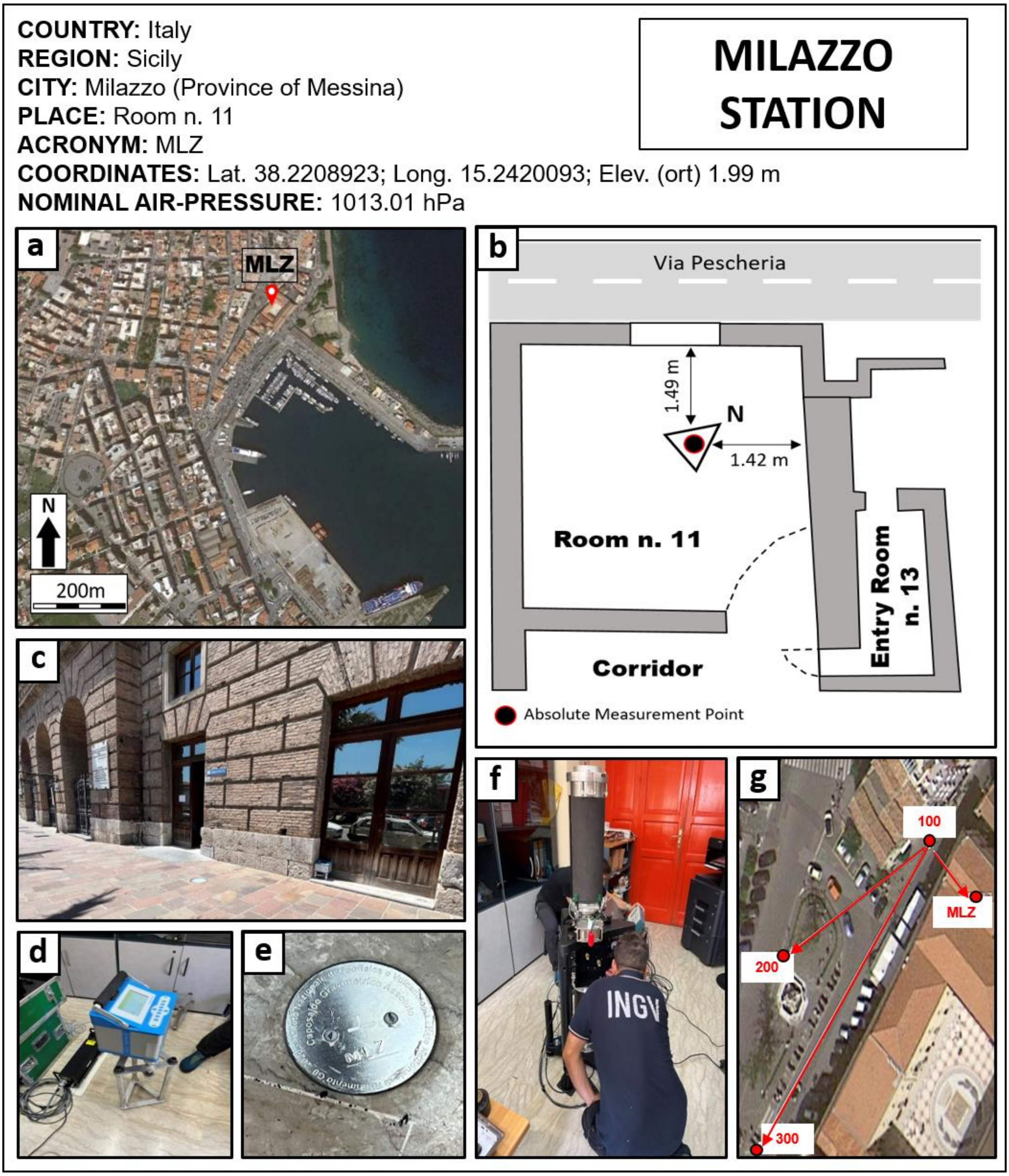


**Fig. A3** Schematic monograph and pictures of the absolute gravity station MLZ. (**a**) Google Earth view of the area where the Milazzo (ME) absolute station is located (coordinates are described at the top of the figure); (**b**) detailed planimetry of the room with the exact location of the absolute measurement point; (**c**) external view of the building and the location of the satellite station, used for fast link with the absolute point; (**d**) the Scintrex CG-6 gravimeter during vertical gravity gradient measurement; (**e**) photo of the stainless steel marker on which the acronym of the station is marked; (**f**) the Micro-g LaCoste FG5#238 during the measurement session; (**g**) Milazzo surveyed network (MLZ - absolute gravity point, 100 - Total Station, 200 and 300 - outdoor reference points (GNSS))

**COUNTRY:** Italy
**REGION:** Sicily
**CITY:** Noto (Province of Siracusa)
**PLACE:** Istituto Nazionale di Astrofisica
**ACRONYM:** NTO
**COORDINATES:** Lat. 36.8760409; Long. 14.9890523; Elev. (ort) 83.42 m
**NOMINAL AIR-PRESSURE:** 1003.27 hPa

**NOTO STATION**

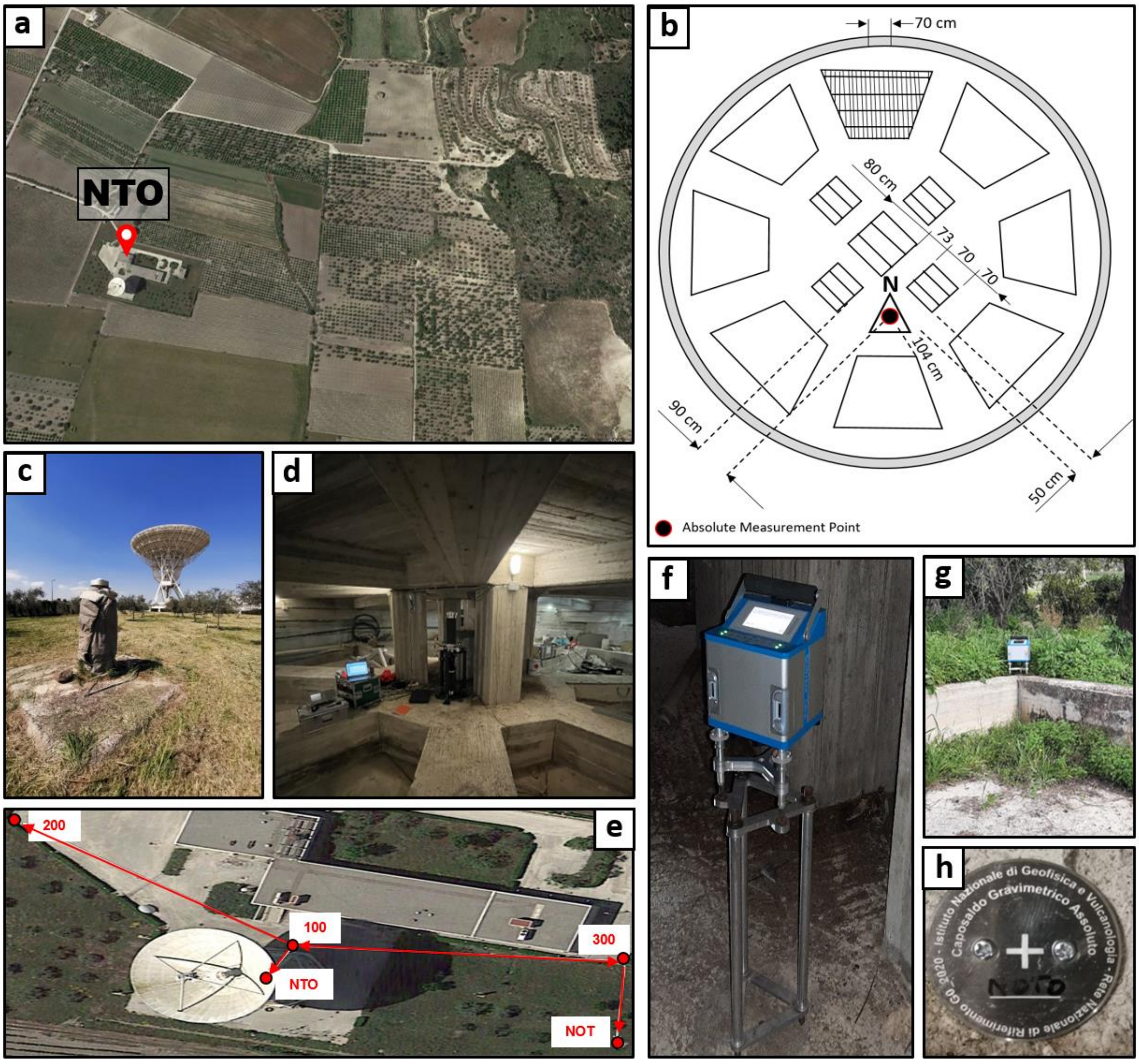


**Fig. A4** Schematic monograph and pictures of the absolute gravity station NTO. (**a**) Google Earth view of the area where the Noto (SR) absolute station is located (coordinates are described at the top of the figure); (**b**) detailed planimetry of the room with the exact location of the absolute measurement point; (**c**) external view of the building where g measurement occurred; (**d**) The Micro-g LaCoste FG5#238 during the measurement session; (**e**) NOTO surveyed network (NTO - absolute gravity point, 100 - Total Station, 200 and 300 - outdoor reference points (GNSS), NOT1 - GNSS Permanent Station; (**f**) the Scintrex CG-6 during vertical gravity gradient measurement; (**g**) measurement location of the external satellite station, used for fast link with the absolute point; (**h**) photo of the stainless steel marker on which the acronym of the station is marked

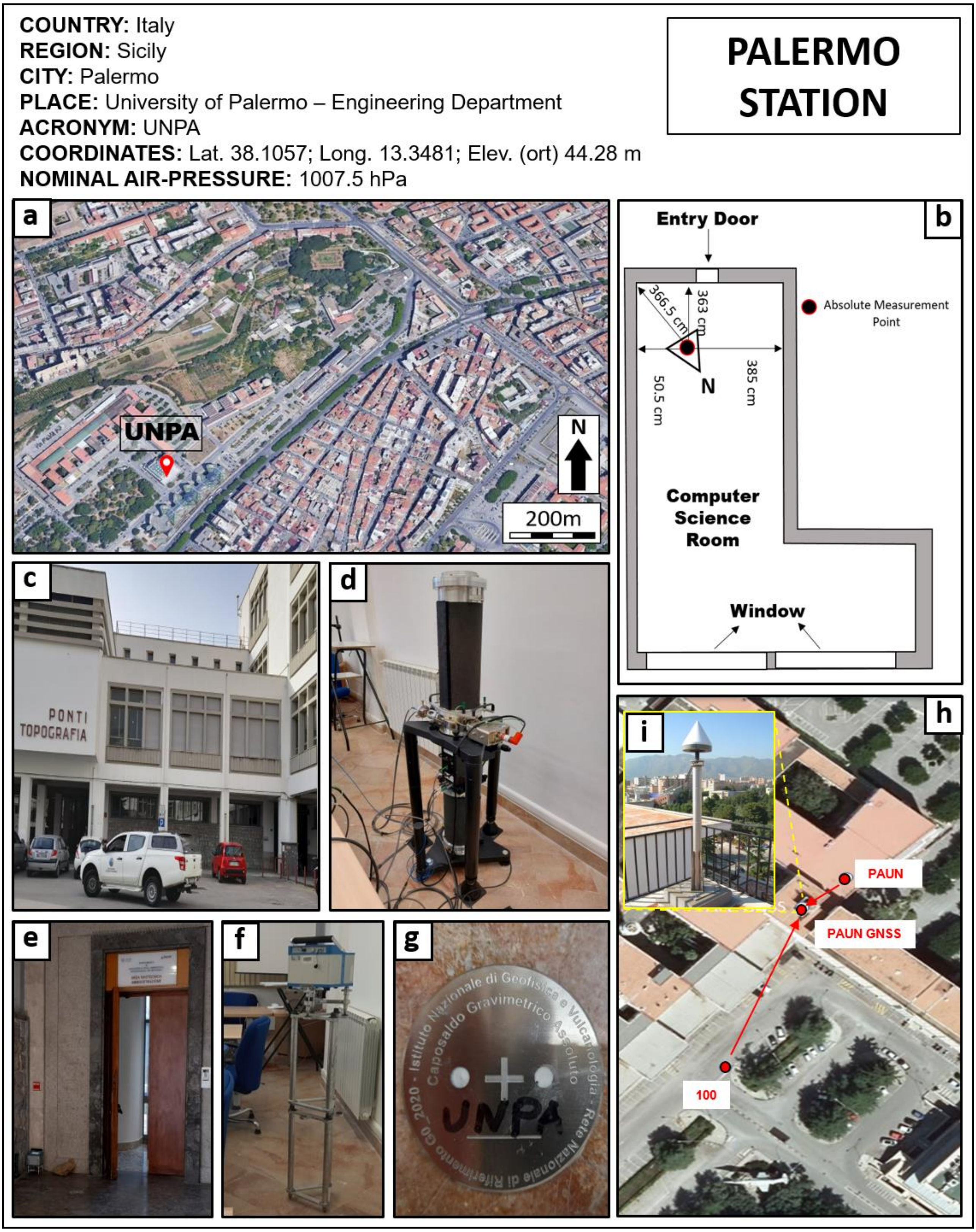


**Fig. A5** Schematic monograph and pictures of the absolute gravity station UNPA. (**a**) Google Earth view of the area where the Palermo absolute station is located (coordinates are described at the top of the figure); (**b**) detailed planimetry of the room with the exact location of the absolute measurement point; (**c**) external view of the building where g measurement occurred; (**d**) the Micro-g LaCoste FG5#238 during the measurement session; **e** external relative satellite station (UNPA_S1); (**f**) the Scintrex CG-6 during vertical gravity gradient measurement. (**g**) photo of the stainless steel marker on which the acronym of the station is marked; (**h**) PAUN surveyed network (PAUN - Point on the roof along the vertical where g was measured, 100 - Total Station and digital level, PAUN GNSS – RDN GNSS Permanent Station); (**i**) Particular of the GNSS PAUN station.

Supplementary information
The online version contains supplementary material and database available at repository data DOI https://zenodo.org/record/8365542. (open access).
